# Lymphocyte to White Blood Cell Count Ratio an Independent Risk Factor for Heart Failure

**DOI:** 10.3390/life14101266

**Published:** 2024-10-05

**Authors:** Lior Charach, Avishay Spitzer, Lior Zusmanovitch, Gideon Charach

**Affiliations:** 1 Division of Gastroenterology, Laniado Medical Center, Netanya 4244916, Israel; 2 Oncology Institute, Tel Aviv Sourasky Medical Center, Tel Aviv University, Tel Aviv 6423906, Israel; 3 Meuhedet Health Service, Tel-Aviv 6473813, Israel; 4 Holon Institute of Technology Israel, Holon 5810201, Israel

**Keywords:** lymphocyte to white blood cell ratio, heart failure, biomarkers

## Abstract

Objective: Heart failure affects 1–2% of the population in developed countries. Hemogram biomarkers are cheap, rapid, readily accessible and are known to have prognostic benefit in cardiovascular, infectious and oncologic diseases. Methods: The aim of the current study is to evaluate lymphocyte-to-white-blood-cell ratio (LWR) as a prognostic predictor in patients with heart failure. Patients with heart failure were recruited between January 2000 and July 2001. Exclusion criteria included metastatic malignancy, exposure to chemotherapy, radiotherapy or medications known to affect complete blood count. Results: 338 patients were enrolled, 33 were excluded. Mean age was 70.1 ± 10.8, 225 patients were male (73%) and 80 were female (27%). All patients were divided into three groups according to LWR. Group 1 < 0.2, group 2—0.2 < LWR < 0.35 and group 3 > 0.35. Patients with LWR ratio < 0.2 had the poorest survival while patients in the highest LWR (ratio > 0.35) had the best long-term survival. Conclusions: Patients with congestive heart failure and LWR < 0.2 showed significant increased mortality. LWR was shown as independent prognostic predictor for HF patients compared to other main outcome parameters, including CRP, NYHA, EF and LDL.

## 1. Introduction

Inflammation plays a crucial role in the atherogenic process and can lead to cardiovascular sequela including ischemic heart disease and heart failure (HF) [1,2]. Non-ischemic cardiomyopathy (NICM) may lead to heart failure and known etiologies of NICM include genetic, infiltrative, medications, radiation, viruses, parasites, post-partum and valvular diseases [3].

Heart failure affects approximately 65 million people worldwide, with a prevalence of 1–2% among the general adult population in developed countries [4]. Heart failure classification had several modifications during the past 25 years [5]. Previously, HF was classified as either systolic or diastolic dysfunction and the current study used this classification because it started at 2000. Since 2005, the American College of Cardiology/American Heart Association guidelines adopted a new classification based on ejection fraction, which is also known as heart failure with reduced ejection fraction (HFrEF) and heart failure with preserved ejection fraction (HFpEF). During 2020, the Heart Failure society of America together with the Heart Failure Association of the European Society of Cardiology and the Japanese Heart Failure Society suggested four different classes according to left ventricular ejection fraction (LVEF): HF with reduced ejection fraction (LVEF ≤ 40%), HF with mildly reduced ejection fraction (LVEF 41–49%), HF with preserved ejection fraction (LVEF ≥ 50%) and a new classification, HF with improved ejection fraction, which is defined as symptomatic HF with LVEF ≤ 40%, a ≥10-point increase from baseline LVEF, and a second measurement of LVEF >40% [5,6].

Cheap, rapid and easily accessible biomarkers are needed to prognosticate heart failure. Hemogram biomarkers raise great interest due to their characteristics which reflect an organism’s health condition. Several studies tested biomarkers as prognostic factors in cardiovascular diseases, including monocytes, lymphocytes and neutrophils. Neutrophil-to-lymphocyte ratio (NLR) has been extensively studied among patients with heart failure, and high NLR was shown to be associated with worse outcomes [7,8,9,10]. Furthermore, platelet-to-lymphocyte ratio (PLR), monocyte-to-lymphocyte ratio (MLR) and lymphocyte-to-monocyte ratio (LMR) were studied in patients with heart failure and are used as prognostic biomarkers [11,12,13,14].

Lymphocytes play a role in systemic inflammatory response and thus contribute to atherosclerosis development [15,16]. Low lymphocyte count is considered as a predictive biomarker of unfavorable outcomes in patients with heart failure, chronic ischemic heart disease and acute coronary syndromes [17,18,19]. Possible etiologies for this finding may be associated with the sympathetic nervous system and renin–angiotensin–aldosterone hormones, which show that elevated angiotensin, cortisol and adrenaline are associated with oxidative stress, proapoptotic effect on lymphocytes and increase in WBC [20,21,22].

Lymphocyte-to-white-blood-cell ratio has been shown to be a prognostic biomarker in infectious diseases such as endocarditis, hepatitis B and COVID-19 as well as in oncological diseases [23,24,25].

The aim of this study is to evaluate the prognostic role of lymphocyte-to-white-blood-cell ratio in patients with congestive heart failure.

## 2. Methods

All patients in this prospective study were successfully recruited from an out-patient heart failure clinic in Tel Aviv Medical Center. The study was approved by the institutional ethics committee (No-0554-17-TLV) and each subject provided informed consent to participate. Patients included were between age 18–95 years with symptoms of congestive heart failure and history of ischemic heart disease who were followed between January 2000 and July 2001. Baseline blood samples were collected, including complete blood count with differential counts, lipid profile and kidney function test. On the first visit, all participants were examined by a physician, medical history was obtained, blood pressure, heart rate and weight were measured, New York Heart Association (NYHA) was determined and trans-thoracic echocardiography was performed. Systolic heart failure was defined as left ventricular ejection fraction less than 40% and diastolic dysfunction was defined as ejection fraction above 40%. Ischemic heart disease was defined according to confirmed myocardial infarction per electrocardiography, cardiac biomarkers including creatine phosphorkinase MB, troponin or both, pathological stress test (thallium or technetium), pathological coronary angiography or coronary artery bypass grafting. Follow-up was at least every quartile.

Exclusion criteria included patients with metastatic malignancy, exposure to chemotherapy, radiotherapy or medications that are known to affect the complete blood count; acute and severe renal failure; active liver disease; severe pulmonary disease; active infectious disease; recent acute myocardial infarction, which was defined as less than 3 months and heart surgery diseases that have an impact on lymphocyte and total leucocyte count.

## 3. Statistical Analyses

All statistical analyses were performed with R version 4.0.3. Survival analysis was performed using the R survival and survminer packages. All reported tests were 2-sided, and *p*  <  0.05 was considered significant.

All continuous variables are displayed as mean (SD). Categorical variables are displayed as numbers (percentages) of participants within each group. Continuous normally distributed variables were compared with a *t*-test, continuous non-normally distributed variable with the Kruskal–Wallis test and categorical variables with the χ2 test. Participants with missing data were excluded from all analyses.

## 4. Patient Assignment to Groups

Patients were classified into three groups based on the ratio of lymphocytes count to WBC ($\frac{Lymphocytes}{WBC}$). This ratio was calculated for each patient, and the distribution of these ratios was then divided into three groups based on the 25th and 75th percentiles of the distribution. Patients with $\frac{Lymphocytes}{WBC}$ ratio falling below the 25th percentile were assigned to the *Low* group, those between the 25th and 75th percentiles to the Medium group, and those above the 75th percentile to the High group. The Medium group was set as the baseline level of the categorical variable. Similarly, the patients were divided into three groups for other measured variables—left ventricular ejection fraction, New York Heart Association creatinine, hemoglobin, total cholesterol, low-density lipoprotein (LDL), high-density lipoprotein (HDL), triglyceride, C-reactive protein (CRP), polymorphonuclear (PMN) to lymphocyte ratio, monocyte to lymphocyte ratio, platelet to lymphocyte ratio, absolute monocyte count, absolute lymphocyte count and N-terminal pro b-type natriuretic peptide (NT-pro-BNP).

## 5. Cox Regression Analysis

Participants were censored on event occurrence (i.e., death) or at the end of the study period. All measured variables and the age and gender covariates were tested with Schoenfeld tests to evaluate the assumption of hazard proportionality, revealing that age was a non-proportional hazard. Then, the effect associated with each measured variable was estimated by generating an age-stratified Cox regression model including the measured variable and gender as a covariate. Finally, the results of all Cox models were pulled into a single table.

Survival curves were estimated with the Kaplan–Meier method for each measured variable independently.

## 6. Results

A total of 338 patients were enrolled in this study, 33 patients were excluded due to lack of follow up, noncompliance or technical reasons. The mean age of the patients that were included in this study was 70.1 ± 10.8 years. The mean follow up was 11.3 years and 78% of the patients necessitated hospitalization during follow-up. A total of 60% of the patients had systolic dysfunction and 40% had diastolic dysfunction.

Table 1 describes general characteristics, clinical and laboratory parameters divided into three lymphocyte/white blood cell groups (group 1 ratio < 0.2, group 2 ratio 0.2–0.35, group 3 ratio > 0.35). Patients in group 1 (<0.2) were older, (mean 74.8 ± 10.6), had higher NYHA classification, higher NT-proBNP levels and lower LDL levels (*p* < 0.001) compared to group 3 (LWR > 0.35). Patients in group 3 (LWR > 0.35) were younger (mean 67.4 ± 9.4), had higher monocyte count, creatinine clearance, LDL count (*p* < 0.001) and lower NT-pro-BNP levels (*p* < 0.001).

**Figure 1:**
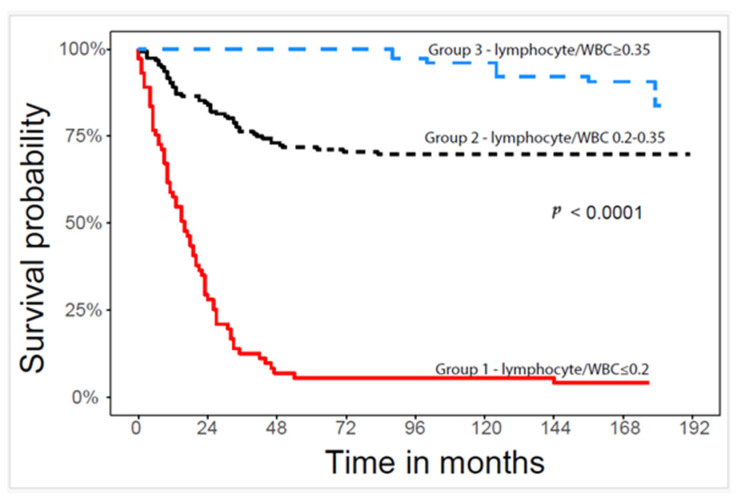
Kaplan–Meier survival curves divided into three groups according to lymphocytes to WBC ratio.

**Figure 2:**
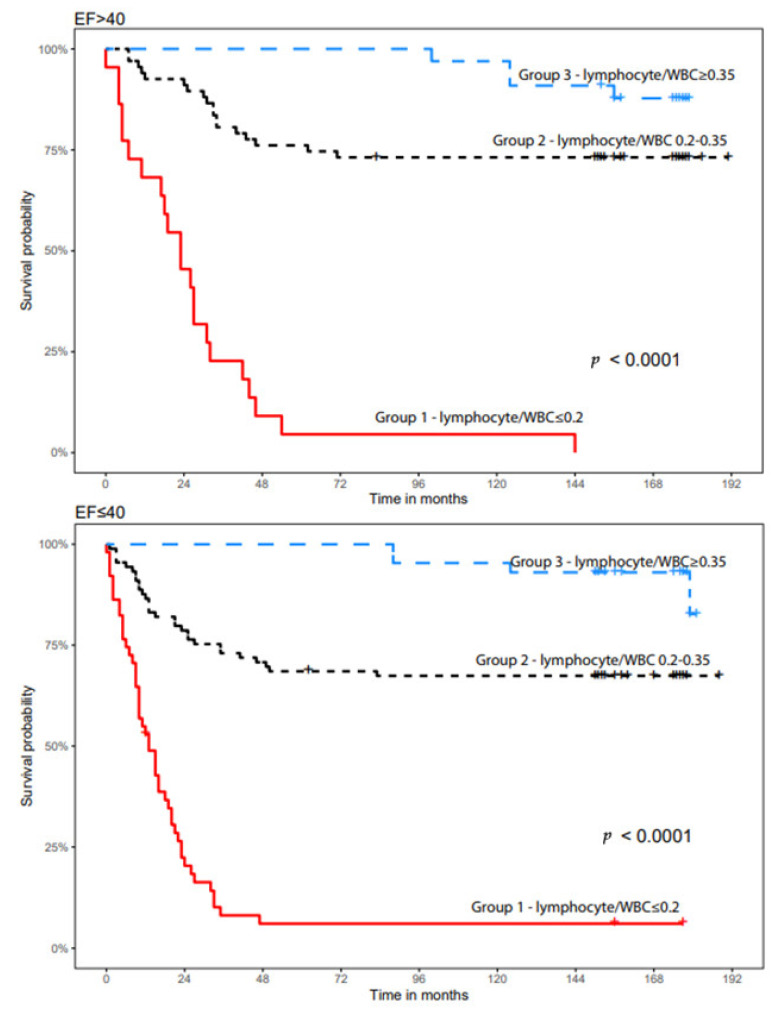
Kaplan–Meier curve according to left ventricular ejection fraction.

**Figure 3:**
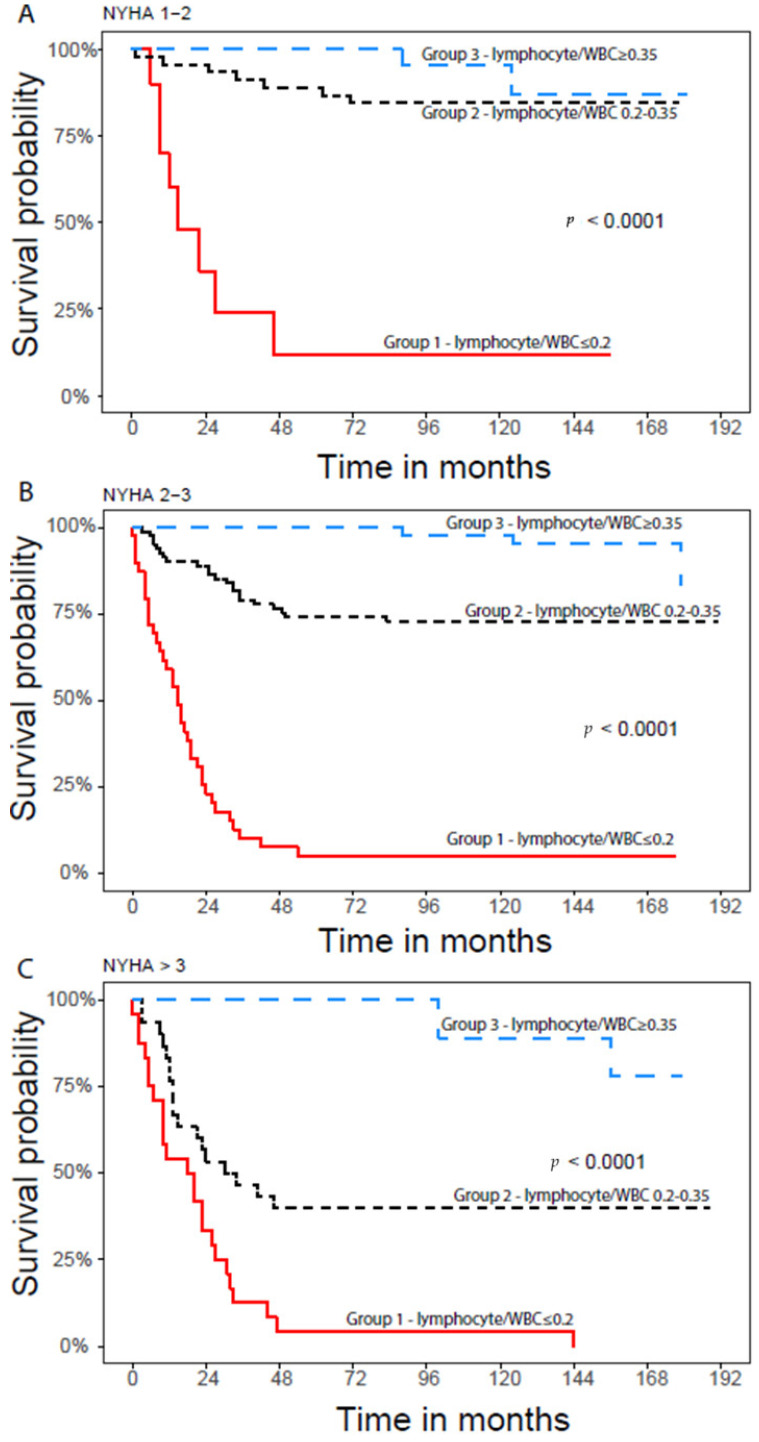
Kaplan–Meier curve according to NYHA class. Figure (**A**) shows survival probability among patients with NYHA classes 1–2; figure (**B**) shows survival probability among patients with NYHA classes 2–3; figure (**C**) shows survival probability among patients with NYHA class above 3.

Kaplan–Meier survival curves divided into three groups of LWR is shown in Figure 1. Patients in group 1 had the poorest survival, 10% for 16 years (192 months), while patients in group 3 demonstrated the best long term survival, 84%. We examined main HF clinical characteristics, such as LVEF and NYHA class according to LWR, and their impact on survival. Figure 2 and Figure 3 present the Kaplan–Meier curve according to LVEF and NYHA class, showing that patients with LVEF above 40% and high LWR had substantial better survival compared to patients with low ratio, 89% vs. 10% respectively (*p* < 0.0001). Patients with LVEF < 40% showed significant difference in survival, patients with high ratio (LWR > 0.35) had 89% (*p* < 0.0001) compared to 9% in the low ratio group. Table 2 shows the hazard ratio (HR) in comparison to group 2 (25th and 75th percentiles). HR was lower in the highest LWR (*p* < 0.05). Patients in group 3 (LWR > 0.35) with NYHA class 1–2 showed better survival compared to group 1, 85% versus 17%, respectively. In NYHA class 3–4 patients in group 3 (LWR > 0.35) showed better survival as well, 84%, versus 5% in group 1 (*p* < 0.0001). These findings demonstrate high predictive value of LWR. Similar results of better outcome and prolonged survival were seen in Kaplan–Meier analysis between LWR, CRP, LDL, creatinine and hemoglobin. The highest HR was seen in LWR ≤ 0.2; moreover, patients with poor cardiac function manifested by NT-pro BNP above 4336 pg/mL, LVEF ≤ 25% and NYHA 3 had high HR as well. Polymorphonuclear (PMN) to lymphocyte ratio ≥ 3.2 also exhibited high HR and can partially be explained by the high neutrophil fraction in white blood cell count (WBC). Further clinical and demographic parameters including hypertension, hemoglobin, HDL and LDL did not show statistical significance. Pearson’s correlation index analysis did not show any correlation between LWR and mentioned variables. Chi Square analysis assessed the dependency of the categorical variables and LWR was shown to be independent prognostic parameter when compared with CRP, NYHA, EF and LDL.

## 7. Discussion

Lymphocytes are a valuable component in the inflammatory process and low levels are associated with increased risk of atherosclerosis and its sequela, such as heart failure and ischemic heart disease [15,16,17,18,19]. Previous studies evaluated different hemogram components and ratios in relation to atherosclerosis and heart failure. High neutrophil counts and neutrophil-to-lymphocyte ratio were associated with increased atherosclerotic cardiovascular risk and mortality among patients with heart failure [26,27,28,29]. Low lymphocytes count was shown to be associated with increased mortality in patients with acute decompensated heart failure [30]. Monocytes count was considered as a useful predictive biomarker and indicator of unfavorable outcomes among patients with acute coronary syndrome, post-infarction, heart failure, coronary artery disease and atherosclerosis [7,8]. Previous studies showed a relationship between total WBC, including eosinophil, neutrophil, and monocyte counts separately. Total WBC counts were related to the severity of coronary artery disease, and higher WBC counts increased the risk of cardiovascular diseases [31,32].

One pathophysiological explanation for these observations can be the activation of the renin-angiotensin system and adrenergic nervous system, which leads to elevated stress hormones and to programmed cell death in lymphocytes. These hormonal changes lead to an increase in neutrophil count and to relative lymphocytopenia [20,21,22]. Moreover, oxidative stress was shown to be associated with the activation of inflammatory cells, which leads to myocardial damage and thus contributes to heart failure progression [33].

LWR has been studied in association with infectious diseases, including infective endocarditis, COVID-19, acute on chronic liver failure secondary to hepatitis B virus and in patients with cancer [23,24,25,34]. Zhang Y. et al. [23] stated that in patients with acute on chronic liver failure secondary to hepatitis B virus, LWR can be used as an independent risk factor for poor outcomes after 28 days with a cutoff of 0.11 [23]. Zhang M. et al. [24] evaluated the prognostic value of LWR in patients with endocarditis and showed higher mortality among patients with low LWR, with cutoff of 0.1. Zhao et al. [25] showed that, in patients with advanced malignancy receiving palliative care, low LWR is associated with increased hazard ratio for mortality compared to high LWR. Formiga et al. [35] assessed LWR among elderly with first acute HF hospitalization and showed that patients with low LWR had higher mortality rates.

The current study divided patients with heart failure into three groups according to LWR. Patients with lowest LWR, which was set below 0.2, showed significant increased mortality compared to patients with higher LWR. The highest HR, 5.74, was in group 1, with LWR < 0.2 (*p* < 0.001) and the lowest HR, 0.26, was in the group 3, LWR > 0.35. It makes possible to consider that LWR is a simple, low cost and important predictor of outcome in patients with HF. Moreover, LWR can be considered as an independent prognostic predictor when compared to other main outcome parameters, including CRP, NYHA, EF and LDL.

NYHA class was higher in group 1, although LDL was lower (*p* < 0.001). One possible explanation for this finding is cardiac cachexia phenomena, which is seen in advanced heart failure, where high catabolic state is associated with low cholesterol levels and implies poor prognosis [22,36].

Other biomarkers were evaluated as well in this study. CRP was reported to bind to oxidized low-density lipoprotein (OxLDL) as part of the innate immune response to oxidized phosphorylcholine-bearing phospholipids in this modified lipoprotein [18]. In the current study, the HR for CRP above 8.67 showed statistical significance; however, lower values were not statistically significant. This may suggest that, while CRP may be related to myocardial injury, it is not a good predictor for long-time outcomes of HF. This finding is in concordance with previous reports [18,37].

Natriuretic peptides improve discrimination for HF prognosis above conventional risk factors and improve risk classification for heart failure [38]. Previous studies compared CRP to NT-pro BNP or other biomarkers like OxLDL. CRP has prognostic value based on mechanism of inflammation and oxidative stress which occurs in atherosclerosis; however, other biomarkers like NT-proBNP or OxLDL were more sensitive and are associated with better prediction [18]. In this study, total lymphocyte count showed the best correlation and was the best predictor of survival, including NT-pro BNP [18]. Previously, we evaluated the prognostic value of NT-proBNP in a 3.7-year follow-up study in patients with chronic HF [18]. NT-proBNP emerged as potential biomarker of clinical interest in HF management. NT-proBNP is related to HF severity and to clinical status. NT-proBNP was strongly associated with prognosis across the entire spectrum of HF. However, NT-proBNP can be low or normal in balanced HF [29,39].

Vitamin D binding (VDB) protein levels have been shown to correlate with cardiovascular disease, including myocardial infarction and HF. VDB protein was not available at the time of recruitment and there was not enough data about the prognostic effect of VDB protein and HF, thus it was not measured at HF units [40,41].

There were limitations to this study, which included relatively small sample size and enrollment being limited to a single center. We did not evaluate biomarkers such as vascular cell adhesion protein 1, CD34+ cells, endothelial progenitor cells, vascular endothelial growth factor receptor-2, tumor necrosis factor alpha and its receptor and stromal derived factor-1, which are known to increase in HF. Procalcitonin, which also shown prognostic value, and its levels correlate with adverse clinical outcomes and severity was not evaluated, because the information was not available at the study onset. Further prospective, multicenter studies with larger cohort will be needed to establish the conception of LWR as good prognostic predictor in patients with HF.

## 8. Conclusions

This study assesses LWR biomarker in relation to patients with congestive heart failure. LWR is an easily accessible, inexpensive and reliable prognostic biomarker and may help clinicians assessing high-risk patients as early as possible. Patients with congestive heart failure and LWR < 0.2 showed significant increased mortality while patients with LWR > 0.35 expressed better survival. LWR is independent prognostic predictor for HF patients when compared to main outcome parameters including CRP, NYHA, EF and LDL.

## Figures and Tables

**Table 1:** Baseline demographic, clinical and laboratory characteristics according to lymphocyte to WBC groups.

	Group1 LWR < 0.2	Group2 0.2 < LWR < 0.35	Group3 LWR > 0.35	*p*-Value
N	76	158	71	
Age (mean (SD))	74.82 (10.62)	69.12 (10.96)	67.42 (9.46)	<0.001
Male (%)	61 (80.3)	112 (70.9)	52 (73.2)	0.31
LVEF (%) (mean (SD))	34.63 (15.67)	37.94 (14.3)	37.73 (13.29)	0.37
DM (%)	32 (42.1)	67 (42.4)	23 (32.4)	0.328
Smoking (%)	31 (40.8)	40 (25.3)	23 (32.4)	0.053
Hyperlipidemia (%)	44 (57.9)	100 (63.3)	44 (62.0)	0.727
Hypertension (%)	46 (60.5)	89 (56.7)	48 (67.6)	0.296
Ischemic heart disease (%)	58 (76.3)	120 (75.9)	52 (73.2)	0.887
Valve disease (%)	17 (22.4)	30 (19.0)	10 (14.1)	0.432
Weight (kg) (mean (SD))	74.40 (17.44)	77.89 (16.18)	79.62 (14.66)	0.137
LVEF group > 40 (%)	26 (34.2)	68 (43.0)	28 (39.4)	0.432
NYHA (mean (SD))	3.04 (0.64)	2.66 (0.56)	2.69 (0.63)	<0.001
NYHA 1–2	12 (15.8)	44 (27.8)	22 (31.0)	
NYHA > 3	32 (42.1)	17 (10.8)	14 (19.7)	
Follow up time years (mean (SD))	2.29 (3.83)	9.78 (5.49)	12.79 (2.92)	<0.001
Hospitalizations (%)	46 (60.5)	81 (51.3)	49 (69.0)	0.036
Mortality (%)	69 (90.8)	47 (29.7)	8 (11.3)	<0.001
Hemoglobin (g%) (mean (SD))	14.74 (1.41)	12.94 (1.73)	12.61 (1.48)	0.182
Platelets (10^9^/L) (mean (SD))	235.60 (97.16)	227.22 (74.20)	190.78 (74.91)	0.005
White blood cell (10^9^/L) (mean (SD))	10.49 (4.91)	7.45 (1.76)	5.02 (1.18)	<0.001
Lymphocytes (K/μL) (mean (SD))	16.81 (6.06)	29.65 (6.84)	33.95 (6.84)	<0.001
Polymorphonuclear (K/μL) (mean (SD))	70.34 (24.27)	63.69 (16.97)	59.26 (9.71)	0.003
Monocytes (K/μL) (mean (SD))	5.95 (3.82)	10.84 (5.03)	13.96 (5.40)	<0.001
GPT (IU/L) (mean (SD))	23.14 (21.56)	23.28 (15.60)	20.49 (11.75)	0.493
Alkaline phosphate (IU/L) (mean (SD))	73.29 (54.16)	64.32 (39.40)	64.58 (42.38)	0.322
Creatinine (mg/dL) (mean (SD))	2.28 (1.48)	1.74 (1.21)	1.53 (0.60)	<0.001
Cholesterol (mg/dL) (mean (SD))	186.28 (53.73)	185.64 (37.60)	186.08 (44.56)	0.994
LDL (mg/dL) (mean (SD))	121.07 (46.07)	125.10 (33.45)	139.92 (43.21)	0.008
HDL (mg/dL) (mean (SD))	44.71 (11.70)	44.79 (11.51)	44.19 (11.35)	0.933
Triglyceride (mg/dL) (mean (SD))	171.33 (116.18)	160.31 (91.70)	141.13 (63.67)	0.152
CRP mg/dL (mean (SD))	10.43 (15.83)	8.42 (14.91)	5.18 (7.62)	0.074
NT-proBNP (pg/mL) (mean (SD))	5670.62 (7493.15)	3422.98 (5522.27)	2526.21 (3990.99)	0.003
Albumin (g/L) (mean (SD))	39.72 (9.05)	39.29 (9.36)	39.62 (7.40)	0.931

LVEF—Left ventricular ejection fraction; DM—Diabetes mellitus; NYHA—New-York Heart Association; CRP—C-reactive protein; NT-proBNP-N-terminal pro b-type natriuretic peptide; LDL—low density lipoprotein; HDL—high density lipoprotein; GPT—glutamate pyruvate alanine aminotransferase.

**Table 2:** Mortality Hazard ratio of main clinical and laboratory parameters related to heart failure according to variables groups.

Variable Group	HR	*p* Value	conf.low	conf.high
Lymphocytes to WBC ratio ≤ 0.2	5.74	<0.001	3.81	8.66
Lymphocytes to WBC ratio ≥ 0.35	0.26	<0.001	0.12	0.57
LVEF ≤ 25%	1.91	<0.01	1.26	2.91
LVEF ≥ 50%	1.07	0.74	0.68	1.69
NYHA 1–2	0.6	0.05	0.35	1.02
NYHA > 3	1.82	<0.01	1.23	2.7
Creatinine (mg/dL) ≤ 1.2	0.58	0.06	0.32	1.03
Creatinine (mg/dL) ≥ 1.5	1.81	<0.01	1.23	2.66
Hemoglobin ≤ 12 g%	1.15	0.48	0.76	1.74
Hemoglobin ≥ 14 g%	0.76	0.29	0.47	1.25
Total Cholesterol ≤ 162 (mg/dL)	1.43	0.08	0.95	2.16
Total Cholesterol ≥ 203 (mg/dL)	1.1	0.65	0.7	1.75
LDL Cholesterol ≤ 102 (mg/dL)	1.36	0.13	0.9	2.05
LDL Cholesterol ≥ 147 (mg/dL)	0.83	0.44	0.52	1.32
HDL Cholesterol ≤ 36 (mg/dL)	0.79	0.31	0.5	1.24
HDL Cholesterol ≥ 52 (mg/dL)	0.97	0.92	0.64	1.49
Triglycerides ≤ 95 (mg/dL)	1.26	0.27	0.83	1.91
Triglycerides ≥ 195 (mg/dL)	1.33	0.2	0.85	2.08
CRP (mg/dL) ≤ 1.52	0.71	0.17	0.44	1.15
CRP (mg/dL) ≥ 8.67	1.72	<0.01	1.15	2.57
PMN to lymphocytes ratio ≤ 1.75	0.21	<0.001	0.09	0.5
PMN to lymphocytes ratio ≥ 3.2	3.34	<0.001	2.27	4.92
Monocytes to lymphocytes ratio ≤ 0.27	2.01	<0.001	1.36	2.97
Monocytes to lymphocytes ratio ≥ 0.47	0.56	0.02	0.33	0.94
Platelets to lymphocytes ratio ≤ 80	0.32	<0.001	0.17	0.6
Platelets to lymphocytes ratio ≥ 154	2.07	<0.001	1.41	3.04
NT-proBNP ≤ 653 (pg/mL)	0.55	0.04	0.3	0.99
NT-proBNP ≥ 4336 (pg/mL)	2.29	<0.001	1.56	3.35

LVEF—Left ventricular ejection fraction; NYHA—New-York Heart Association Functional Class; PMN—polymorphonuclear; CRP-C–reactive protein; NT-proBNP—N-terminal pro b-type natriuretic peptide.

## Data Availability

Available data can be made public, in accordance with the journal’s policy.

## References

[1] Frąk W., Wojtasińska A., Lisińska W. (2022). Pathophysiology of cardiovascular diseases: New insights into molecular mechanisms of atherosclerosis, arterial hypertension, and coronary artery disease. Biomedicines.

[2] Björkegren J.L., Lusis A.J. (2022). Atherosclerosis: Recent developments. Cell.

[3] Fayol A., Wack M., Livrozet M. (2022). Aetiological classification and prognosis in patients with heart failure with preserved ejection fraction. ESC Heart Fail..

[4] Groenewegen A., Rutten F.H., Mosterd A. (2020). Epidemiology of heart failure. Eur. J. Heart Fail..

[5] Lam C.S., Yancy C. (2021). Universal definition and classification of heart failure: Is it universal? Does it define heart failure?. J. Card. Fail..

[6] Fonarow G.C. (2017). Refining classification of heart failure based on ejection fraction. JACC Heart Fail..

[7] Curran F.M., Bhalraam U., Mohan M. (2021). Neutrophil-to-lymphocyte ratio and outcomes in patients with new-onset or worsening heart failure with reduced and preserved ejection fraction. ESC Heart Fail..

[8] Benites-Zapata V.A., Hernandez A.V., Nagarajan V. (2015). Usefulness of neutrophil-to-lymphocyte ratio in risk stratification of patients with advanced heart failure. Am. J. Cardiol..

[9] Bhat T., Teli S., Rijal J. (2013). Neutrophil to lymphocyte ratio and cardiovascular diseases: A review. Expert Rev. Cardiovasc. Ther..

[10] Durmus E., Kivrak T., Gerin F. (2015). Neutrophil-to-lymphocyte ratio and platelet-to-lymphocyte ratio are predictors of heart failure. Arq. Bras. Cardiol..

[11] Naylor S. (2003). Biomarkers: Current perspectives and future prospects. Expert Rev. Mol. Diagn..

[12] Shahid F., Lip G.Y., Shantsila E. (2018). Role of monocytes in heart failure and atrial fibrillation. J. Am. Heart Assoc..

[13] Wong K.L., Yeap W.H., Tai J.J.Y. (2012). The three human monocyte subsets: Implications for health and disease. Immunol. Res..

[14] Vakhshoori M., Nemati S., Sabouhi S. (2023). Selection of monocyte-to-lymphocyte ratio (MLR) or lymphocyte-to-monocyte ratio (LMR) as best prognostic tool in heart failure: A systematic review. SN Compr. Clin. Med..

[15] Frostegård J. (2013). Immunity, atherosclerosis and cardiovascular disease. BMC Med..

[16] Hedrick C.C. (2015). Lymphocytes in atherosclerosis. Arterioscler. Thromb. Vasc. Biol..

[17] Núñez J., Miñana G., Bodí V. (2011). Low lymphocyte count and cardiovascular diseases. Curr. Med. Chem..

[18] Charach G., Grosskopf I., Roth A. (2011). Usefulness of total lymphocyte count as predictor of outcome in patients with chronic heart failure. Am. J. Cardiol..

[19] Acanfora D., Gheorghiade M., Trojano L., CHF Italian Study Investigators (2001). Relative lymphocyte count: A prognostic indicator of mortality in elderly patients with congestive heart failure. Am. Heart J..

[20] Marra S., Hoffman-Goetz L. (2004). β-adrenergic receptor blockade during exercise decreases intestinal lymphocyte apoptosis but not cell loss in mice. Can. J. Physiol. Pharmacol..

[21] Abrams M.T., Robertson N.M., Yoon K. (2004). Inhibition of glucocorticoid-induced apoptosis by targeting the major splice variants of BIM mRNA with small interfering RNA and short hairpin RNA. J. Biol. Chem..

[22] Anker S.D., Chua T.P., Ponikowski P. (1997). Hormonal changes and catabolic/anabolic imbalance in chronic heart failure and their importance for cardiac cachexia. Circulation.

[23] Zhang Y., Chen P., Zhu X. (2023). Lymphocyte-to-white blood cell ratio is associated with outcome in patients with hepatitis B virus-related acute-on-chronic liver failure. World J. Gastroenterol..

[24] Zhang M., Ge Q., Qiao T. (2022). Prognostic Value of Lymphocyte-to-White Blood Cell Ratio for In-Hospital Mortality in Infective Endocarditis Patients. Int. J. Clin. Pract..

[25] Zhao W., Wang P., Jia H. (2017). Lymphocyte count or percentage: Which can better predict the prognosis of advanced cancer patients following palliative care?. BMC Cancer.

[26] Luo J., Thomassen J.Q., Nordestgaard B.G. (2023). Neutrophil counts and cardiovascular disease. Eur. Heart J..

[27] Vakhshoori M., Nemati S., Sabouhi S. (2023). Neutrophil to lymphocyte ratio (NLR) prognostic effects on heart failure; a systematic review and meta-analysis. BMC Cardiovasc. Disord..

[28] Charach G., Rogowski O., Karniel E. (2019). Monocytes may be favorable biomarker and predictor of long-term outcome in patients with chronic heart failure: A cohort study. Medicine.

[29] Silva N., Bettencourt P., Guimarães J.T. (2015). The lymphocyte-to-monocyte ratio: An added value for death prediction in heart failure. Nutr. Metab. Cardiovasc. Dis..

[30] Uthamalingam S., Patvardhan E.A., Subramanian S. (2011). Utility of the neutrophil to lymphocyte ratio in predicting long-term outcomes in acute decompensated heart failure. Am. J. Cardiol..

[31] Kim J.H., Lim S., Park K.S. (2017). Total and differential WBC counts are related with coronary artery atherosclerosis and increase the risk for cardiovascular disease in Koreans. PLoS ONE.

[32] Kounis N.G., Soufras G.D., Tsigkas G. (2015). White blood cell counts, leukocyte ratios, and eosinophils as inflammatory markers in patients with coronary artery disease. Clin. Appl. Thromb./Hemost..

[33] Aimo A., Castiglione V., Borrelli C. (2020). Oxidative stress and inflammation in the evolution of heart failure: From pathophysiology to therapeutic strategies. Eur. J. Prev. Cardiol..

[34] Pitre T., Jones A., Su J. (2021). Inflammatory biomarkers as independent prognosticators of 28-day mortality for COVID-19 patients admitted to general medicine or ICU wards: A retrospective cohort study. Intern. Emerg. Med..

[35] Formiga F., Chivite D., Salvatori M. (2018). Lymphocyte-to-white blood cells ratio in older patients experiencing a first acute heart failure hospitalization. Eur Geriatr Med..

[36] Von Haehling S., Schefold J.C., Springer J. (2008). The cholesterol paradox revisited: Heart failure, systemic inflammation, and beyond. Heart Fail. Clin..

[37] George J., Wexler D., Roth A. (2006). Usefulness of anti-oxidized LDL antibody determination for assessment of clinical control in patients with heart failure. Eur. J. Heart Fail..

[38] Smith J.G., Newton-Cheh C., Almgren P. (2010). Assessment of conventional cardiovascular risk factors and multiple biomarkers for the prediction of incident heart failure and atrial fibrillation. J. Am. Coll. Cardiol..

[39] Anand I.S., Latini R., Florea V.G. (2005). C-reactive protein in heart failure: Prognostic value and the effect of valsartan. Circulation.

[40] Gasparri C., Curcio A., Torella D. (2010). Proteomics reveals high levels of vitamin D binding protein in myocardial infarction. Front Biosci Elite Ed.

[41] Petrone A.B., Weir N.L., Steffen B.T. (2013). Plasma vitamin D-binding protein and risk of heart failure in male physicians. Am. J. Cardiol..

